# Light-triggered Supramolecular Isomerism in a Self-catenated Zn(II)-organic Framework: Dynamic Photo-switching CO_2_ Uptake and Detection of Nitroaromatics

**DOI:** 10.1038/srep34870

**Published:** 2016-10-11

**Authors:** Wei-Chao Song, Xun-Zhe Cui, Zhong-Yi Liu, En-Cui Yang, Xiao-Jun Zhao

**Affiliations:** 1 Key Laboratory of Inorganic-Organic Hybrid Functional Material Chemistry, Ministry of Education, Tianjin Key Laboratory of Structure and Performance for Functional Molecules, Tianjin Normal University, Tianjin 300387, People’s Republic of China; 2 Department of Chemistry, Collaborative Innovation Center of Chemical Science and Engineering, Nankai University, Tianjin 300071, People’s Republic of China

## Abstract

A self-catenated Zn(II)-organic framework formulated as [Zn_2_(3,3′-bpeab)(oba)_2_]·DMF (1) exhibiting a six-connected 4^4^·6^10^·8 topology has been successfully synthesized through the mixed-ligand of kinked 3,3′-bis[2-(4-pyridyl)ethenyl]azobenzene (3,3′-bpeab) and 4,4′-oxybis-benzoic acid (H_2_oba) under solvothermal condition. UV light triggers isomerization of complex 1 in a single-crystal-to-single-crystal (SCSC) manner, giving rise to a conformational supramolecular isomer 1_UV through the pedal motion of photoresponsive double bonds. Dynamic photo-switching in the obtained light-responsive supramolecular isomers leads to instantly reversible CO_2_ uptake. Furthermore, the ligand originated fluorescence emission of water-resistant complex 1 is selectively sensitive to 4-nitrotoluene (4-NT) owing to a higher quenching efficiency of the perilous explosive over other structurally similar nitroaromatics, prefiguring the potentials of 1 as a fluorescence sensor towards 4-NT in aquatic media.

Metal-organic frameworks (MOFs) with structural dynamic properties can further achieve the modulation of the network via external stimuli triggered single-crystal to single-crystal (SCSC) transformation, referring to bending, sliding, rotation, distortion, cleavage or formation of coordination bonds^1^,^2^,^3^. Nevertheless, the feasibility of photo- or heat- induced SCSC reactions is undermined by the skeleton deformation of MOFs, placing significance on 3D networks with adequate strength to endure molecules rearrangement without forfeiting crystallinity^4^,^5^,^6^,^7^. Hence great efforts have been devoted to the design and discovery of transformable compounds under physical stimuli. Due to the −N=N− can present *trans*-to-*cis* transformation or C−C−N bending of azo moiety under UV trigger^5^,^8^,^9^,^10^, dynamic MOFs based on azobenzene unit exhibiting photo-switching behaviors have attracted much attention. While a molecular-level understanding of photo-switching CO_2_ uptake in azo based MOFs where azo groups are part of the MOF wall is not yet accessible. Stimulus triggered pedal-like motion of double bonds provides a solution for regio- or stereo- specific compounds to be obtained effortlessly via the reorganization of disordering molecules in crystal lattice. Few examples of SCSC transformations via the pedal motion of olefinic bond or azo moiety of the ligands have been reported^11^,^12^,^13^, no physical responsive MOFs have been constructed from ligands containing both kinds of bonds. In spite of the well-established studies on compounds containing single reactive unit^14^,^15^,^16^,^17^,^18^, it is still a daunting task to construct a suitable system to investigate the mechanism of transformation of multiple responsive groups embedded in one substrate. There should be more examples that can be rationalized on the assumption of pedal motion and the “invisible” conformer.

On the other hand, the facile fluorescence quenching of various electron-deficient nitroaromatic explosives by a charge-transfer mechanism bestows sensitivity, convenience and swiftness upon photoluminescence sensing MOFs in its detection, but also poses a challenge to selective detection of particular nitro analyte^19^. In addition, the hydrolytic stability of the MOF holds practical significance, while only limited numbers of water resistant MOFs showing chemo-sensing properties have been reported till now^20^,^21^,^22^,^23^. Introducing hydrophobic groups near coordination sites is an effective method to improve water stability of M(II)-carboxylate-based MOFs. In addition, the catenation may improve the water resistance of MOFs for the difficulty in the displacement of the ligands locked within the framework^24^. Pillared-layer MOFs with different degrees of interpenetration have been constructed from mixed-ligand of rigid linear dicarboxylate linkers and diamine ligands. While the construction of self-catenated pillared-layer MOFs from mixed flexible or kinked ligands remains largely unexplored, especially with helical character^25^,^26^.

Herein, a pillar ligand 3,3′-bis[2-(4-pyridyl)ethenyl]azobenzene (3,3′-bpeab) bearing dual distinctive stimuli-responsive functional units (−C=C− and −N=N− bonds) is designed. The combination of step like 3,3′-bpeab and V-shaped 4,4′-oxybisbenzoic acid (H_2_oba) as ligands may favor the formation of diverse and helical structures, and promote the formation of self-catenation, which is beneficial to obtain moisture stable porous frameworks. Fortunately, a self-catenated porous Zn^II^-organic framework based on paddle-wheel type secondary building units (SBUs) of Zn_2_(CO_2_)_4_ was isolated. The incorporation of photo responsive components into the coordination network leads to its dynamic manners, more particularly, the interconversion of conformers of 3,3′-bpeab, which is scarcely practicable through conventional synthetic methods. Fortunately, the crystallinity of resulting crystals upon stimulus is retained to offer complete structural details, which could provide useful insights into the relationship of the photo-switching CO_2_ uptake and the conformational changes. The photoluminescence properties of long delocalized 3,3′-bpeab are enhanced as rigidifying the aromatic conjugated ligand into the Zn(II) porous framework results in a non-radiative relaxation reducing, allows the π-electron rich framework a considerable candidate for fluorescence sensing.

## Results

### Synthesis

Solvothermal reaction of Zn(NO_3_)_2_·6H_2_O, 3,3′-bpeab and H_2_oba gives crystals of **1**. Same patch of crystals was received UV irradiation to obtain complex **1_UV**. Single crystals of **1** were heated at 100 °C for 2 hours under vacuum to get complex **1_heat**.

### Structure description of [Zn_2_(3,3′-bpeab)(oba)_2_]·DMF (1)

**Figure 1:**
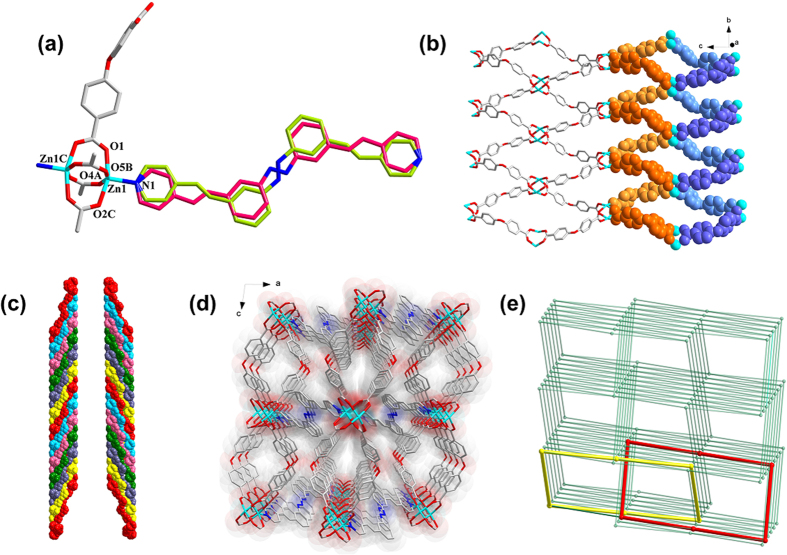
(**a**) Coordination environments of the metal ions and ligands in complex **1** (H atoms were omitted for clarity, distinct colors are used for two conformations). (**b**) The 2D sub-network bridged by oba ligands in **1**. (**c**) A pair of sextuple-stranded helices along the *b* direction (different colors are used for the six strands). (**d**) A perspective view of the 3D framework along the *b*-axis. (**e**) The 3D self-penetrating framework with a uninodal 6-connected (4^4^.6^10^.8) topology (two shortest six-member rings are catenated) considering the binuclear SBUs as nodes.

Complex **1** crystallizes in the monoclinic space group *C*2/*c*. The asymmetric unit contains one crystallographically independent Zn^II^ ion, half a 3,3′-bpeab ligand, one oba ligand and half a guest DMF solvent molecule. Structural studies indicated that the metal center possesses a tetragonal pyramid geometry, coordinated by one pyridinic nitrogen atom from the 3,3′-bpeab ligand and four oxygen atoms from four different oba ligands (Fig. 1a). The Zn−O bond lengths fall in the range of 2.030–2.046 Å and Zn−N bond lengths range from 2.000 to 2.013 Å. It is worth mentioning that, the 3,3′-bpeab ligand displays two distinct conformations with different occupancies, nearly 53.6% of which adopts conformation I and the remaining 46.4% adopts the conformation II, due to the different orientations of the −C=C− and −N=N− bonds (Figure S1). The two terminal pyridyl rings in the bpeab ligand are both coplanar with respect to the middle phenyl ring. Zn1 and Zn1A are linked by four bridging carboxyl bridges to form a dinuclear paddle-wheel Zn_2_(COO)_4_ secondary building unit (SBU). The SBUs are further connected to four more units by the oba ligands to obtain a 2D [Zn_2_(oba)_2_] sublayer lying in the *bc* plane with rhombic grids (Fig. 1b). The 2D layer is developed by two kinds of helical chains running along the *b*-axis with a pitch of 9.798 Å, where the right- and left-handed helical chains with the same composition of (-Zn-oba-Zn-oba-)_*n*_ are aligned in an alternate array by sharing the Zn(II) centers, leaving a mesomeric 1D channel with interchanging chiralities (Fig. 1b). These highly corrugated (4, 4) 2D layers are further pillared by 3,3′-bpeab ligands to construct a pillared-layer porous 3D framework (Figs 1d and S2). Unlike the typical parallel arrangement, adjacent bpeab ligands employ a criss-cross manner, coupling with the oba ligands to generate unusual intertwined sextuple-stranded helical chains in the *b*-axis direction (Fig. 1c).

Topologically, each dinuclear SBU is connected to six identical units, four by individual oba ligands, and the other two by 3,3′-bpeab ligands; thus the Zn_2_(COO)_4_ SBUs can be simplified as six-connected nodes, and the whole structure can be described as a six-connecting uninodal net with Schlafli symbol of 4^4^.6^10^.8 (TD_10_ = 6679) (Fig. 1e). It is proverbial that the commonly encountered single pillared-layer structures generally exhibit pcu topology with different interpenetrations when utilize linear dicarboxylate as ligands. While the flexible oba ligands can tune the orientation around the paddlewheel SBUs to form different sub-layers, which was further pillared by bipyridyl-based ligands with distinct topologies, and several cases with roa, jsm, 6T9 topologies have been reported recently^27^,^28^,^29^,^30^,^31^. It should be noted that, the 6T9 topology and the observed topology of complex **1** have the same Schlafli symbol, but with different TD_10_ (5391 for 6T9). In the 6T9 topology, interpenetrated 2D double layers are pillared by the pyridyl spacer ligands, while the current observed topology of complex **1** is constructed from non-interpenetrated single layers. In addition, an interesting feature of this topology is the presence of self-catenation. The extremely tight self-catenation causes a high topological density of the net, TD_10_ = 6679, as each 6-ring is crossed by 132 other rings (50 6-rings and 82 8-rings). According to the RCSR database^32^, this is the highest topological density among all known 6-coordinated nets. Two smallest six-membered circuits form the catenane-like interlocking structure as highlighted in Figs 1e and S3. The six-membered circuit consists of a pair of bpeab, four oba ligands and six Zn^II^ dimers, with the distances of 26.778 and 27.825 Å between two neighbouring vertices, bringing about a 68.45° intersection angle between the two edges (Figure S4).

### The SCSC transformation of complex 1 via stimulus of UV light

**Figure 2:**
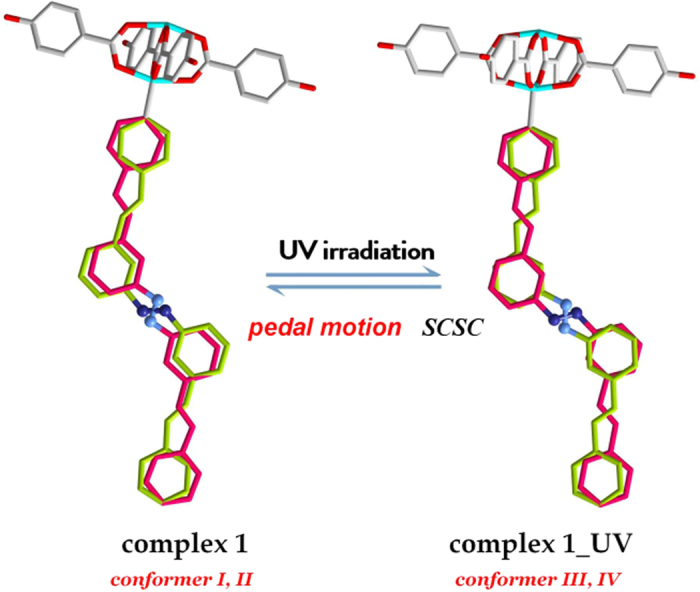
Light-triggered SCSC transformation through pedal motion in 3,3′-bpeab.

Pedal motion has been observed in compounds with −C=C− bonds just as those with −N=N− bonds, extending the scope of this movement to various molecules such as azobenzenes, stilbenes, etc^12^,^33^,^34^,^35^,^36^. In the present study, the 3,3′-bpeab molecule couples two types of selected pedal motion groups, −C=C− double bonds and −N=N− moiety, to explore the pedal motion in crystals under stimulus of UV light. The UV irradiated samples **1_UV** remained the same connectivity with complex **1** (Figure S5). Compared with complex **1**, the distance of the dinuclear SBUs across 3,3′-bpeab in **1_UV** varied from 26.778 Å to 26.923 Å as 3,3′-bpeab ligands converted to conformation III and VI, different from both of the two conformations in parent crystal, as depicted in Figs 2 and S5. While the distance of the dinuclear SBUs through oba ligand varies along with the −C−O−C− angular distortion of the ligand from 13.966 Å to 14.079 Å, and 117.27° to 118.45°, respectively (Figure S6), besides, slight deformation of the rhombic grids also leads to the interior angle shrinking from 41.07° to 40.55°. The salient feature of complex **1_UV** is −N=N− pedal motion, while whether the −C=C− pedal motion has or how occurred is still inexplicit. In consideration of molecules are not restricted to their lowest energy conformer during crystallization, when external stimulus was introduced to overcome the obstacles of activation energy for isomerization, a series of conformation interchanging was initiated (Figure S7). While irradiated with UV light, the coexisting conformer I, II can transform to conformer III and IV. It is well established that the −N=N− bonds incline to undergo light-induced reversible *trans*-to-*cis* isomerization, while such a transformation was suppressed in the coordination framework, resulting in the pedal motion of azo moiety under UV light. Although the −C=C− pedal motion is not observed in the UV irradiation process through the X-Ray diffraction, the probable transformation process may involve the isomerization of −C=C− bonds in conformer I towards conformer IV or conformer II to conformer III, respectively. In addition, the 3,3′-bpeab molecules are disposed in a slip-stacked manner in complex **1**, leaving phenyls closer to the adjacent pyridine rings with a 4.038 Å distance among the parallel aligning −C=C− bond pairs. Despite satisfying the Schmidt’s criteria, [2 + 2] photochemical cycloaddition reaction was excluded, this might ascribe to the pedal motion of the double bonds. It should be noted that not all the feasible mechanism for such conformational changes have been included. The possibilities of a certain synergism or interplay between all the aforementioned isomerization processes have not been ruled out.

### The SCSC transformation of complex 1 via stimulus of heat

After **1** was heated at 100 °C under vacuum for 2h without losing its single crystallinity (with some cracks), X-ray diffraction analysis reveals that the structure held the same connectivity with small deviation in relative positions of Zn atoms to form **1_heat**, as the volume of the unit cell was decreased from 5755(10) Å^3^ to 5648.3(9) Å^3^, which might be affected by the removal of the solvent molecules (Figure S8). The Zn−Zn distance through 3,3′-bpeab and oba are also changing to 23.626 Å and 14.171 Å, respectively. The most obvious conversion of the 3,3′-bpeab ligands was the −N=N− bonds pedal motion since the conformations of −C=C− bonds appear to maintain in the heating process. The −N=N− bonds change to a same orientation unanimously, leaving the 3,3′-bpeab ligands as conformer II and III. Along with the pedal motion, the inside acute angle of the six-membered metallocyclic ring widens even more to 69.05°, with concomitant variation of the rhombus [Zn_8_(oba)_4_] ring as its inside acute angle shrinks even more to 39.75°, comparing to the ones in complexes **1** and **1_UV**. The different existing conformers in complexes **1_UV** and **1_heat** show different ways of transformation among conformers triggered by the stimulus of UV or heat.

### Thermal Stability and Moisture Stability

The TG analysis curve for complex **1** shows a weight loss of about 6.7% near 150 °C, corresponding to the loss of the solvent DMF molecules (Figure S9). And the TGA spectrum of **1_activated** shows a plateau before its collapse, suggesting the complete removal of the solvent molecules that occupied the frameworks. After sample of **1** was immersed in water over a week, the obtained sample shows one-step weight loss process until the decomposition temperature at 320 °C. The PXRD patterns of the activated, water immersed samples, water boiled samples coincide with the simulated one, suggesting the stability towards temperature and humidity (Figure S10). As far as we know, there are rare MOFs showing good stability in boiling water^17^,^37^,^38^. Structurally, the acute angle of the adjacent phenyls around the SBUs is smaller due to the bent nature of the oba ligand, and the distance between the adjacent H atoms of phenyl of oba and the pyridinyl of the 3,3′-bpeab ligand is smaller than the usually observed distance in pillared-layer MOFs based on the Zn_2_-paddle-wheel SBUs (Figure S11), which may enhance the shielding ability of the ligand to protect the SBUs against water molecules. In addition, the self-catenation of the framework is another key factor to its moisture resistance^24^.

### Porosity Measurements and Photo Switching Studies

**Figure 3:**
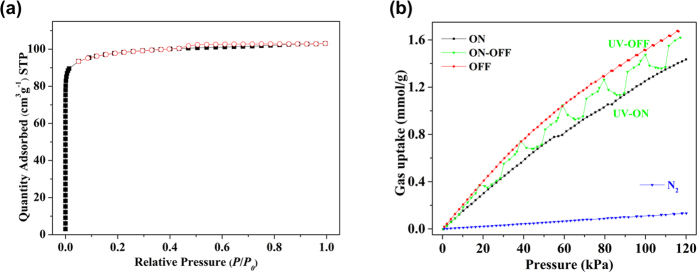
(**a**) N_2_ isotherms at 77 K for **1**. (**b**) CO_2_ adsorption isotherms of complex **1** at 298 K in the absence of light (black), presence of light (red), and light switching conditions (green); N_2_ adsorption isotherms at 298 K (blue).

The high water stability and the azo decorated porous structures, make the complex a potential candidate for gas separation under practical conditions. The dynamic nature of the double bonds under UV light may induce the structural flexibility to influence the interactions with CO_2_ molecules. In addition, the channels are decorated by the O atoms of the oba ligand and N atoms of the 3,3′-bpeab ligands, which may facilitate the interactions with CO_2_ molecules to resulting a higher uptake than other gases, which is a prerequisite for the application of a separation material. The isotherm of activated **1** at 77 K shows a normal type-I isotherm shape, indicative of permanent micropores, resulting in BET surface areas of 299 m^2^/g (Fig. 3a). As shown in Fig. 3b, the activated **1** adsorbs very small amounts of N_2_, while the uptake of CO_2_ at 120 kPa is ~13 times higher than N_2_. Selectivity is of fundamental importance in processes such as gas separation, the near-linear adsorption profiles for N_2_ is indicative of their low affinity with the frameworks as expected from its relatively low polarizability. The Henry’s constants were employed to estimate the selectivity of the complex. **1** shows ideal CO_2_/N_2_ adsorption selectivity of 19.7 at 298 K. The activated **1** can absorb substantial amounts of CO_2_ with the uptake capacity of 1.69 mmol/g at 298 K and 120 kPa. These values are comparable to the best performing ZIF material, but are moderate compared to some highly porous MOFs due to the much lower surface area of **1**^39^,^40^,^41^. Isosteric adsorption enthalpies (*Q*_st_) as a function of the quantity of gases adsorbed were calculated using virial method (Figure S12). Virial analysis shows that the enthalpies of CO_2_ adsorption is 28.3 kJ/mol. Such a moderate *Q*_st_ value is a strong advantage for the implementation of low-energy regeneration for CO_2_ separation. In addition, keeping physicochemical stability is the primary consideration for practical applications, many MOFs face the hydrolysis issues and restrict their application in humid conditions, because of the dative nature of the metal-ligand bonds. The boiling water treatment of the sample results in lower crystallinity and partial collapse of the framework, the N_2_ uptake at 77 K is negligible, however, the CO_2_ uptake is 0.63 mmol/g at 298 K and 120 kPa, about 37% of the pristine sample (Figure S13).

Under static irradiation conditions, the CO_2_ uptake capacity drops to 1.43 mmol/g at 298 K and 120 kPa (Fig. 3b). The dynamic irradiation isotherms follow values obtained under continuous irradiation conditions or UV-OFF conditions. In order to clarify whether the irradiation would promote the formation of a steady state of the conformations, the sample was irradiated under UV light for 3 hours before the adsorption experiment (Figure S14). And the isotherms follow values collected under no UV light. The phenomenon indicates the flexible nature of the framework can be triggered by UV irradiation, and the transformation of the conformers occurred in a dynamic fashion. The UV−vis spectrum exhibits two absorption bands in the UV region at 297 nm and 382 nm, which are attributed to π–π* and n–π* electronic transitions (Figure S15). Small fractions of the structure were found to periodically oscillate under irrdiation, this may be ascribed to the rotation of the phenyl ring and the bending movement of the related bonds. The transformations are occurred quite quickly in the UV-vis experiment under UV light. In addition, the gas adsorption experiment also indirectly provides a view of this phenomenon, the gas uptake can trace the switching of the light. A different batch of sample was collected again under the same photo-switching experiment, and the results show subtle difference, probably due to the different exposing surface area of the samples under UV light (Figure S14). The conversion occurred to the powder form that used in the UV−vis experiment is comparable, the single crystals used for gas sorption experiments also change ever so promptly, being able to keep pace with the switching on-and-off of UV light immediately. As stated by Hill *et al*.^9^, light irradiation increased the MOF surface energy to weaken the interactions between CO_2_ molecules, which was correlated with structural oscillations from C−C−N bending movement under UV trigger of the azo-MOF. In the present work, the variations of the CO_2_ uptake may be ascribed to the conformational changes through the pedal motion of the double bonds which induced dynamic flexibility of the framework under UV light.

### Detection of Nitroaromatic Explosives

**Figure 4:**
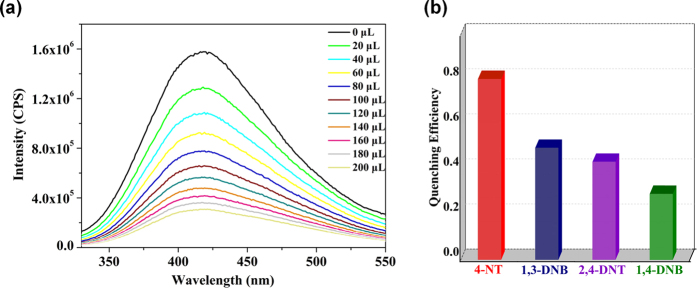
(**a**) Emission spectra of **1** upon incremental addition of saturated aqueous solutions of 4-NT. (**b**) Fluorescence quenching efficiency of **1** dispersed in water upon addition of 200 μL of saturated aqueous solutions of 4-NT, 1,3-DNB, 1,4-DNB, 2,4-DT.

Complex **1** has been shown to retain its crystallinity nature when it was dispersed in H_2_O, the photo luminescence spectra show a maximum at 406 nm, and the intensities are much stronger than other ten more solvents (Figure S16). Due to this emissive property, **1** was tested for sensing some nitro derivatives in water solution, as it is very crucial to test out nitroaromatic explosives using a simple and rapid method for applications such as security-screening, mine-fields analysis and environmental monitoring. The experimental data on water solubility of nitro compounds was obtained from the literature^42^. Fluorescence quenching titrations with different 4-NT addition levels were conducted with an excitation wavelength of 285 nm at room temperature (Fig. 4a). With the addition of 4-NT saturated aqueous solution to a 1 g/L dispersion of **1** in 3 mL water, the fluorescence emissions from **1** are instantly decayed, quenching approximately 80.04% of the initial fluorescence intensity. The fluorescence quenching efficiency could be analyzed by the Stern-Volmer equation, 
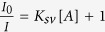
, where *I*_*0*_ and *I* represent the fluorescence intensities of **1** suspension before and after the addition of analyte, respectively; [A] is the concentration of analyte; and *K*_sv_ is the Stern−Volmer quenching constant (ppm^−1^). The quenching constant of *K*_sv_ is an important parameter to describe the fluorescence quenching efficiency, which for 4-NT is quantified to be 8.06 × 10^−2^ ppm^−1^ (Figure S17). Furthermore, to check the potentials of **1** as a fluorescent probe for specific detection, we tested the fluorescence variations of **1** in the presence of various possible nitroaromatic explosives such as 1,3-DNB, 1,4-DNB and 2,4-DNT, all can also act as fluorescence quenchers for **1**, yet even with rather similar chemical structures, their fluorescence quenching efficiencies are much lower than that of 4-NT. The order of *K*_sv_ values for the four quenchers is 4-NT >2,4- DNT >1,4-DNB >1,3-DNB (Figure S18–S20), and the *K*_sv_ values lie in the normal range for the known MOFs^43^,^44^,^45^,^46^. The ratio between *K*_sv_ of 4-NT and that of other nitro explosives is defined as the selective factor (SF), which is generally used to evaluate the selectivity. The SF values for 2,4- DNT, 1,4-DNB, 1,3-DNB over 4-NT are 0.641, 0.331 and 0.256, respectively, suggesting that **1** has a certain degree of selectivity towards 4-NT detection in aqueous solution (Fig. 4b).

## Discussion

To summarize, a six-connected self-catenated Zn(II)-organic framework constructed from 3,3′-bpeab bearing dual distinctive stimuli-responsive functional units (−C=C− and −N=N− bonds) has been successfully synthesized. Dynamic photo-switching in the obtained light-responsive supramolecular isomers leads to instantly reversible CO_2_ uptake, which is ascribed to the light-triggered pedal motion of the double bonds of the 3,3′-bpeab ligands. In addition, complex **1** was tested for sensing a couple of nitro explosives, which displays selective fluorescence quenching towards 4-NT compared to its analogues.

## Methods

### General

All chemicals were commercially purchased and used as received without further purification. The ligand 3,3′-bis[2-(4-pyridyl)ethenyl]azobenzene (3,3′-bpeab) was synthesized according to the literature method^33^. Powder X-ray diffraction (PXRD) patterns were obtained using a Bruker D8 ADVANCE diffractometer at 40 kV and 40 mA for Cu Kα radiation (λ = 1.5406 Å), with a scan speed of 0.1 s per step and a step size of 0.01° in 2θ. The simulated PXRD patterns were calculated using single-crystal X-ray diffraction data and processed by the free Mercury program provided by the Cambridge Crystallographic Data Center. Elemental analyses for C, H and N were determined on a Perkin-Elmer 2400C elemental analyzer. Fourier transform (FT) IR spectra (KBr pellets) were taken on an Avatar-370 (Nicolet) spectrometer. UV-vis spectra (solid) were recorded on a Hitachi U-4100 UV-Vis-NIR spectrophotometer. Thermogravimetric analysis (TGA) experiments were performed on Shimadzu simultaneous DTG-60A compositional analysis instrument from room temperature to 800 °C under N_2_ atmosphere at a heating rate of 10 °C/min. The sorption isotherms for CO_2_ and N_2_ were measured using an automatic volumetric adsorption apparatus (Micrometrics ASAP 2020M). Ultrahigh-purity-grade CO_2_ and N_2_ were used for all measurements. For photo-switching experiments, the UV lamp was surrounded by a cooling system and fixed the sample tube with a distance more than 30 cm to eliminate possible temperature effect on CO_2_ adsorption resulted from UV, and the gas sorption experiments were carried out on intermittently or continuously exposing samples under UV light.

### Preparation of [Zn_2_(3,3′-bpeab)(oba)_2_]·DMF (1)

A mixture of 3,3′-bpeab (19.4 mg, 0.05 mmol), oba (25.8 mg, 0.1 mmol), and Zn(NO_3_)_2_·6H_2_O (29.7 mg, 0.1 mmol) in 5 mL DMF was sealed in a 23 mL Teflon lined stainless steel container and heated at 120 °C for 2 days. After the container was cooled to room temperature, orange block-shaped crystals suitable for X-ray analysis were obtained, washed with DMF, and dried in air (Yield: 70% based on 3,3′-bpeab). Anal. Calcd for C_57_H_43_N_5_O_11_Zn_2_: C, 61.97%; H, 3.92%; N, 6.34%. Found: C, 61.52%; H, 4.21%; N, 6.47%. FT-IR (KBr, cm^−1^): 2926 (w), 1669 (m), 1634 (s), 1609 (s), 1595 (s), 1500 (m), 1394 (s), 1229 (s), 1158 (m), 1087 (w), 1029 (w), 958 (w), 875 (m), 782 (s), 763 (m), 657 (m).

### Preparation of [Zn_2_(3,3′-bpeab)(oba)_2_]·DMF (1_UV)

Single crystals of **1_UV** were obtained by UV irradiation of single crystals of **1**. Anal. Calcd for C_57_H_43_N_5_O_11_Zn_2_: C, 61.97%; H, 3.92%; N, 6.34%. Found: C, 61.63%; H, 4.05%; N, 6.18%. FT-IR (KBr, cm^−1^): 2923 (w), 1674 (s), 1637 (s), 1607 (s), 1499 (m), 1400 (s), 1236 (s), 1159 (m), 1089 (w), 1029 (w), 961 (w), 873 (m), 782 (m), 691 (w), 655 (m).

### Preparation of [Zn_2_(3,3′-bpeab)(oba)_2_] (1_heat)

Single crystals of **1** were heated at 100 °C for 2 hours under vacuum.

### Characterization

Diffraction intensities for **1**, **1_UV** and **1_heat** were collected on Bruker APEX-II CCD diffractometer equipped with graphite-monochromated Mo Kα radiation with radiation wavelength 0.71073 Å by using the φ-ω scan technique at 296 K. Semiempirical multi-scan absorption corrections were applied by *SADABS*^47^, and the program *SAINT*^48^ was used for integration of the diffraction profiles. The structures were solved by direct methods and refined with the full-matrix least-squares technique using the *SHELXS-97* and *SHELXL-97* programs^49^,^50^. Non-H atom was located by difference Fourier maps and subjected to anisotropic refinement. H atom was added according to theoretical models. The crystallographic data and the selected bond lengths and angles were given in Table S1–S4. CCDC 1483995-1483996 and 1503837 contains the supplementary crystallographic data for this paper. These data can be obtained free of charge from the Cambridge Crystallographic Data Centre via https://summary.ccdc.cam.ac.uk/structure-summary-form.

## Additional Information

**How to cite this article**: Song, W.-C. *et al*. Light-triggered Supramolecular Isomerism in a Self-catenated Zn(II)-organic Framework: Dynamic Photo-switching CO_2_ Uptake and Detection of Nitroaromatics. *Sci. Rep*. **6**, 34870; doi: 10.1038/srep34870 (2016).

## Supplementary Material

Supplementary Information
