# Supplementary material for: Light-triggered Supramolecular Isomerism in a Self-catenated Zn(II)-organic Framework: Dynamic Photo-switching CO2 Uptake and Detection of Nitroaromatics
Source: Sci Rep. 2016 Oct 11;6:34870. doi: 10.1038/srep34870 (PMC5057147; doi:10.1038/srep34870)
Supplement: Supplementary Information [file srep34870-s1.pdf]

Electronic Supplementary Information

Light-triggered Supramolecular Isomerism in a Self-catenated Zn(II)-organic Framework: Dynamic Photo-switching CO_2 Uptake and Detection of Nitroaromatics

Wei-Chao Song,[1] Xun-Zhe Cui,[1] Zhong-Yi Liu,[1] En-Cui Yang,[1,]* & Xiao-Jun Zhao[1,2,]*

[1]College of Chemistry, Key Laboratory of Inorganic-Organic Hybrid Functional Material Chemistry, Ministry of Education, Tianjin Key Laboratory of Structure and Performance for Functional Molecules, Tianjin Normal University, Tianjin 300387, People's Republic of China.

[2]Department of Chemistry, Collaborative Innovation Center of Chemical Science and Engineering, Nankai University, Tianjin 300071, People's Republic of China.

*Corresponding Author, Email: encui_yang@163.com and xiaojun_zhao15@163.com

Table of Contents

Table S1. Crystal data and structure refinement for complexes **1**, **1_UV** and **1_heat** ------------------------- 3

Table S2. Selected bond lengths (Å) and angles (°) for complex **1** -- 4

Table S3. Selected bond lengths (Å) and angles (°) for complex **1_UV** --- 5

Table S4. Selected bond lengths (Å) and angles (°) for complex **1_heat** --------------------------------------- 6

Figure S1. Four 3,3'-bpeab ligand conformations observed in the present work ------------------------------ 7

Figure S2. Space-filling diagram of 1D channel in **1** viewed along the *b*-axis --------------------------------- 8

Figure S3. Two catenane-like interlocking six-membered metallocyclic grids -------------------------------- 9

Figure S4. Perspective view of the six-membered metallocyclic grids of (a) **1**; (b) **1_UV**; (c) **1_heat** ---- 10

Figure S5. Coordination environments of the metal ions and ligands in complex **1_UV** -------------------- 11

Figure S6. Perspective view of the rhombus grid of (a) **1**; (b) **1_UV**; (c) **1_heat** ---------------------------- 12

Figure S7. Possible light-triggered pedal motion process of the double bonds -------------------------------- 13

Figure S8. Coordination environments of the metal ions and ligands in complex **1_heat** --------------------13

Figure S9. The TGA curves of **1**, **1**_activated and **1**_water -- 14

Figure S10. PXRD patterns of complex **1** upon treatment with heat, water, boiling water and UV light ---15

Figure S11 Distances of adjacent H atoms of the Zn_2-paddle-wheel SBUs ----------------------------------- 16

Figure S12. (a) Virial expression fitting graphs for CO_2 adsorption data for **1** at 273K (black) and 298K (red). (b) Isosteric heat of CO_2 adsorption for **1** -- 18

Figure S13. N_2 sorption (77 K) and CO_2 sorption (298 K) for boiling treatment samples of **1** ------------- 18

Figure S14. (a) Comparison of the CO_2 uptake at different experimental conditions. (b) Photoswitching experiment of a different batch of sample --- 19

Figure S15. UV-vis spectra under UV from 0 to 60 min --- 19

Figure S16. Emission intensity of **1** at different solvents -- 20

Figure S17 Stern-Volmer plot of F_0/F vs. 4-NT concentration in aqueous solution for **1** ------------------- 20

Figure S18. (a) Emission spectra of **1** at different 1,3-DNB concentrations, (b) Stern-Volmer plot of F_0/F vs. 1,3-DNB concentration in aqueous solution for **1** -- 21

Figure S19. (a) Emission spectra of **1** at different 1,4-DNB concentrations, (b) Stern-Volmer plot of F_0/F vs. 1,4-DNB concentration in aqueous solution for **1** -- 22

Figure S20. (a) Emission spectra of **1** at different 2,4-DNT concentrations, (b) Stern-Volmer plot of F_0/F vs. 2,4-DNT concentration in aqueous solution for **1** -- 23

Table S1. Crystal data and structure refinement for complexes 1, 1_UV and 1_heat.

Crystal	**1**	**1_UV**	**1_heat**
empirical formula	$C_{57}H_{43}N_5O_{11}Zn_2$	$C_{57}H_{43}N_5O_{11}Zn_2$	$C_{27}H_{18}N_2O_5Zn$
F_w	1104.70	1104.70	515.80
crystal size [mm]	0.22 × 0.21 × 0.18	0.22 × 0.21 × 0.18	0.22 × 0.21 × 0.18
crystal syst	Monoclinic	Monoclinic	Monoclinic
space group	*C*2/*c*	*C*2/*c*	*C*2/*c*
a [Å]	23.01(2)	22.782(5)	22.164(2)
b [Å]	9.672(10)	9.758(2)	9.6360(9)
c [Å]	26.09(3)	26.413(5)	26.653(2)
a [°]	90	90	90
β [°]	97.62(2)	97.28(3)	97.127(2)
g [°]	90	90	90
V [Å^3]	5755(10)	5824(2)	5648.3(9)
Z	8	8	8
h / *k* / *l*	-28, 28/ -11, 8/ -32, 32	-27, 27 / -11, 11 / -31, 31	-27, 27 / -10, 12 / -33, 26
F(000)	2272	2272	2112
reflections collected / unique	17602 / 5963	207455 / 5094	18097 / 5851
R_{int}	0.1337	0.1593	0.0437
data / restraints / params	3963 / 326 / 359	5094 / 456 / 359	5851 / 26 / 314
R_1 ^{a}, wR_2 ^{b} [$I > 2\sigma(I)$]	0.0995 / 0.2691	0.1157 / 0.2399	0.0498 / 0.1313
R_1, wR_2 [all data]	0.1543 / 0.3041	0.2422 / 0.2975	0.0790 / 0.1426
GOF on F^2	1.030	1.090	1.054
Δr_{max}, Δr_{min} / e•Å^{-3}	1.012 / -0.761	0.777 / -0.497	0.467 / -0.404

^{a} $R_1 = \Sigma||F_o||F_c||/|F_o|$. ^{b} $wR_2 = [\Sigma w(F_o^2F_c^2)^2/\Sigma w(F_o^2)^2]^{1/2}$.

Table S2. Selected bond lengths (Å) and angles (°) for complex **1**.

Zn(1)–N(1')	1.999(6)	O(4)$^{\#1}$–Zn(1)–O(1)	89.4(2)
Zn(1)–O(2)$^{\#3}$	2.032(5)	N(1')–Zn(1)–O(5)$^{\#2}$	101.2(4)
Zn(1)–O(4)$^{\#1}$	2.029(6)	O(2)$^{\#3}$–Zn(1)–O(5)$^{\#2}$	89.3(2)
Zn(1)–O(1)	2.045(5)	O(4)$^{\#1}$–Zn(1)–O(5)$^{\#2}$	158.3(2)
Zn(1)–O(5)$^{\#2}$	2.030(5)	O(1)–Zn(1)–O(5)$^{\#2}$	99.1(4)
Zn(1)–N(1)	2.012(5)	O(2)$^{\#3}$–Zn(1)–O(1)	158.2(2)
N(1')–Zn(1)–O(2)$^{\#3}$	99.9(4)	O(2)$^{\#3}$–Zn(1)–N(1)	100.0(3)
N(1')–Zn(1)–O(4)$^{\#1}$	100.4(4)	N(1)–Zn(1)–O(4)$^{\#1}$	102.5(4)
O(2)$^{\#3}$–Zn(1)–O(4)$^{\#1}$	86.2(2)	O(1)–Zn(1)–N(1)	101.8(3)
N(1')–Zn(1)–O(1)	102.0(4)	O(5)$^{\#2}$–Zn(1)–N(1)	99.1(4)

[a] Symmetry codes: $^{\#1}$ x, -y + 2, z - 1/2; $^{\#2}$ -x + 1/2, y + 1/2, -z + 1/2; $^{\#3}$ -x + 1/2, -y + 5/2, -z; $^{\#4}$ -x, -y, -z; $^{\#5}$ x, -y + 2, z + 1/2; $^{\#6}$ -x + 1/2, y - 1/2, -z + 1/2.

Table S3. Selected bond lengths (Å) and angles (°) for complex **1_UV**.

Zn(1)–O(2)$^{\#3}$	2.042(6)	O(4)$^{\#1}$–Zn(1)–O(1)	98.5(6)
Zn(1)–O(1)	2.053(7)	N(1)–Zn(1)–O(5)$^{\#2}$	103.2(6)
Zn(1)–N(1)	2.025(12)	O(2)$^{\#3}$–Zn(1)–O(5)$^{\#2}$	89.7(3)
Zn(1)–O(4)$^{\#1}$	2.034(7)	O(4)$^{\#1}$–Zn(1)–O(5)$^{\#2}$	158.2(3)
Zn(1)–O(5)$^{\#2}$	2.037(7)	O(1)–Zn(1)–O(5)$^{\#2}$	86.0(3)
Zn(1)–N(1')	2.080(10)	O(4)$^{\#1}$–Zn(1)–N(1')	103.1(5)
O(4)$^{\#1}$–Zn(1)–O(1)	90.0(3)	N(1)–Zn(1)–O(2)$^{\#3}$	101.6(8)
O(5)$^{\#2}$–Zn(1)–N(1')	98.6(5)	O(2)$^{\#3}$–Zn(1)–O(1)	158.7(3)
O(2)$^{\#3}$–Zn(1)–N(1')	100.2(6)	O(2)$^{\#3}$–Zn(1)–O(4)$^{\#1}$	86.3(3)
O(1)–Zn(1)–N(1')	101.1(6)	N(1)–Zn(1)–O(1)	99.7(8)

[a] Symmetry codes: $^{\#1}$ x, -y + 1, z + 1/2; $^{\#2}$ -x + 1/2, y - 1/2,-z + 1/2; $^{\#3}$ x, -y, z - 1/2; $^{\#4}$ 1/2 - x, 1/2 + y, 1/2 - z; $^{\#5}$ x, -y, z + 1/2; $^{\#6}$ 1 - x, 2 - y, -z.

Table S4. Selected bond lengths (Å) and angles (°) for complex **1_heat**.

Zn(1)–O(2)$^{\#2}$	2.040(2)	O(4)$^{\#3}$–Zn(1)–O(1)	101.4(2)
Zn(1)–O(1)	2.052(2)	N(1)–Zn(1)–O(5)$^{\#1}$	100.2(2)
Zn(1)–N(1)	2.026(3)	O(2)$^{\#2}$–Zn(1)–O(5)$^{\#1}$	86.10(10)
Zn(1)–O(4)$^{\#3}$	2.041(2)	O(4)$^{\#3}$–Zn(1)–O(5)$^{\#1}$	158.41(10)
Zn(1)–O(5)$^{\#1}$	2.039(2)	O(1)–Zn(1)–O(5)$^{\#1}$	90.46(10)
Zn(1)–N(1')	2.040(4)	O(4)$^{\#1}$–Zn(1)–N(1')	101.1(3)
O(4)$^{\#3}$–Zn(1)–O(1)	85.26(10)	N(1)–Zn(1)–O(2)$^{\#2}$	103.6(2)
O(5)$^{\#1}$–Zn(1)–N(1')	100.5(3)	O(2)$^{\#2}$–Zn(1)–O(1)	158.58(10)
O(2)$^{\#2}$–Zn(1)–N(1')	99.6(3)	O(2)$^{\#2}$–Zn(1)–O(4)$^{\#3}$	90.19(10)
O(1)–Zn(1)–N(1')	101.8(3)	N(1)–Zn(1)–O(1)	97.8(2)

^{a} Symmetry codes: $^{\#1}$ x, -y + 1, z - 1/2; $^{\#2}$ -x + 1/2, y + 3/2, -z; $^{\#3}$ -x + 1/2, y + 1/2, -z + 1/2; $^{\#4}$ -x + 1/2, y - 1/2, -z + 1/2; $^{\#5}$ x, -y + 1, z + 1/2; $^{\#6}$ -x + 1, -y - 1, -z.

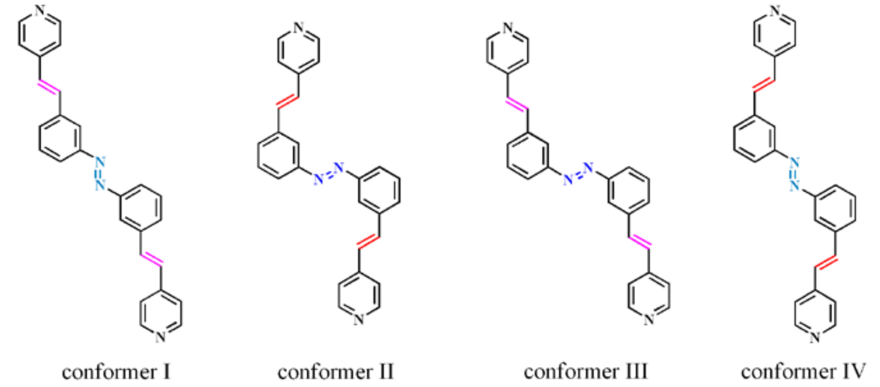

Figure S1 Four conformations of 3,3'-bpeab ligand observed in the present work.

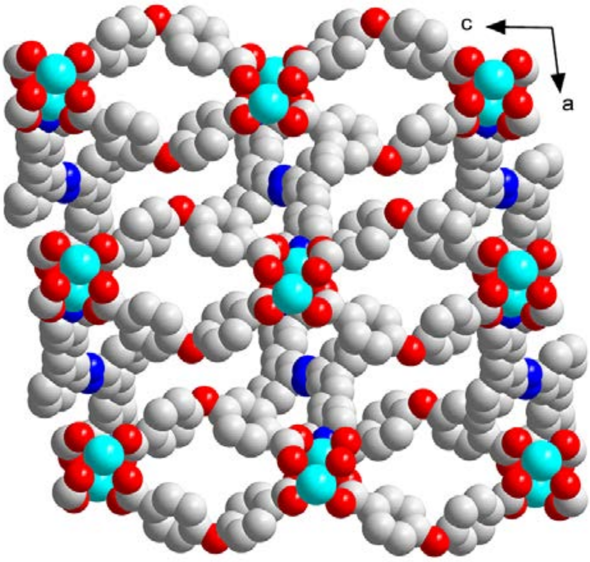

Figure S2 Space-filling diagram of 1D channel in **1** viewed along the *b*-axis.

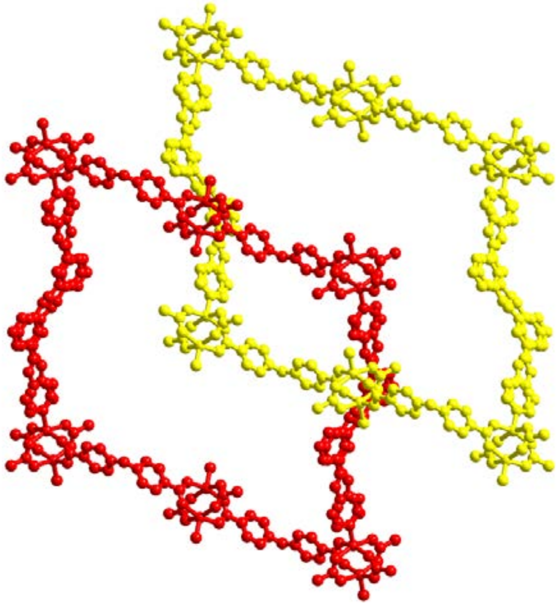

Figure S3 Two catenane-like interlocking six-membered metallocyclic grids.

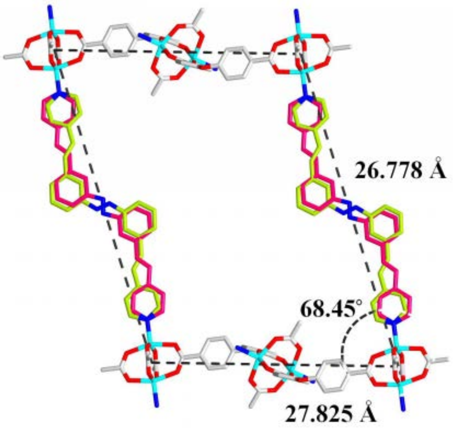

(a)

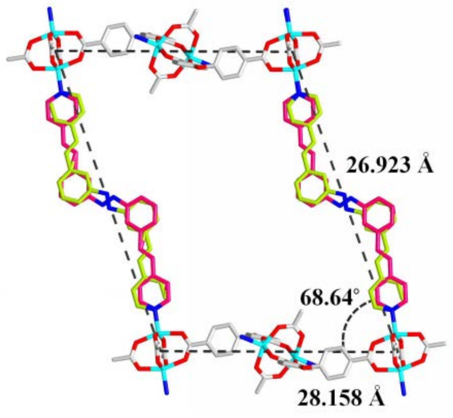

(b)

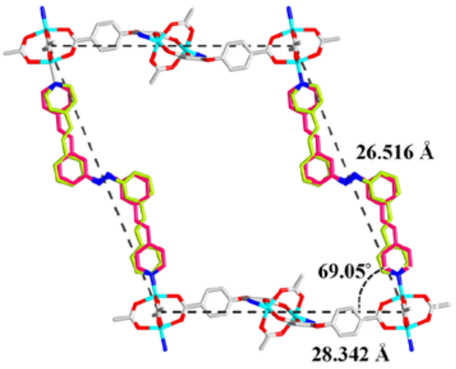

(c)

Figure S4 Perspective view of the six-membered metallocyclic grids of (a) **1**; (b) **1_UV**; (c) **1_heat**.

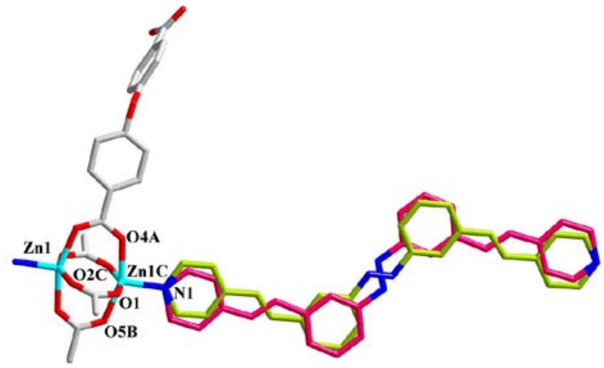

Figure S5 Coordination environments of the metal ions and ligands in complex **1_UV**.

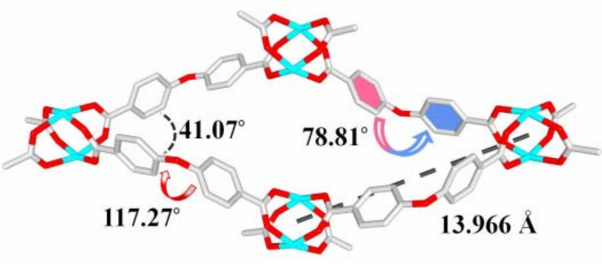

(a)

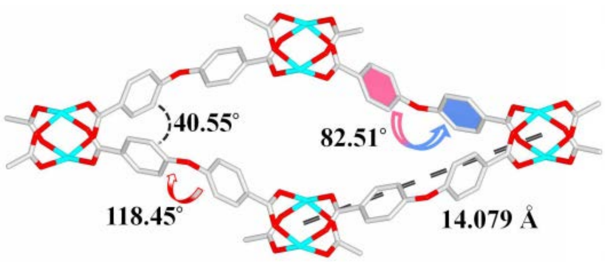

(b)

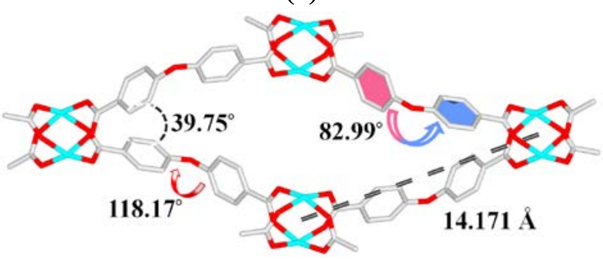

(c)

Figure S6 Perspective view of the rhombus grid of (a) **1**; (b) **1_UV**; (c) **1_heat**.

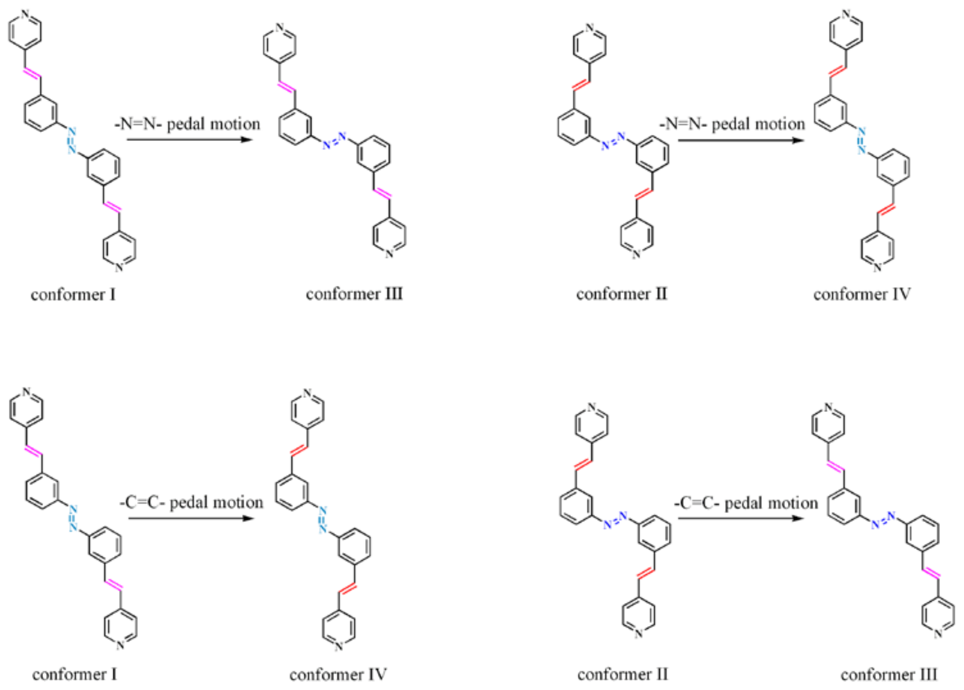

Figure S7 Possible light-triggered pedal motion process of the double bonds.

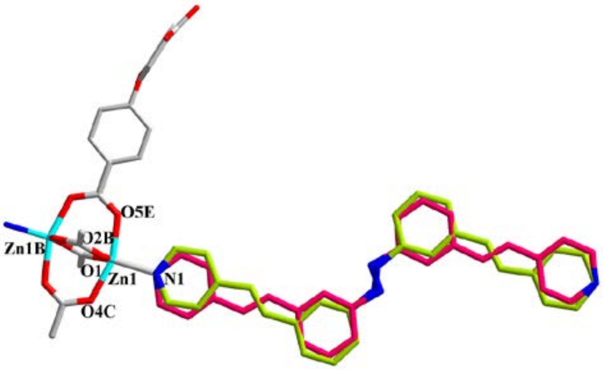

Figure S8 Coordination environments of the metal ions and ligands in complex **1_heat**.

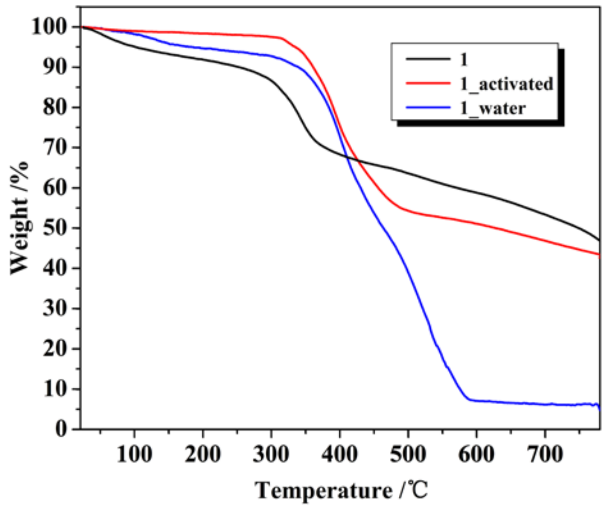

Figure S9 The TGA curves of **1**, **1**_activated and **1**_water.

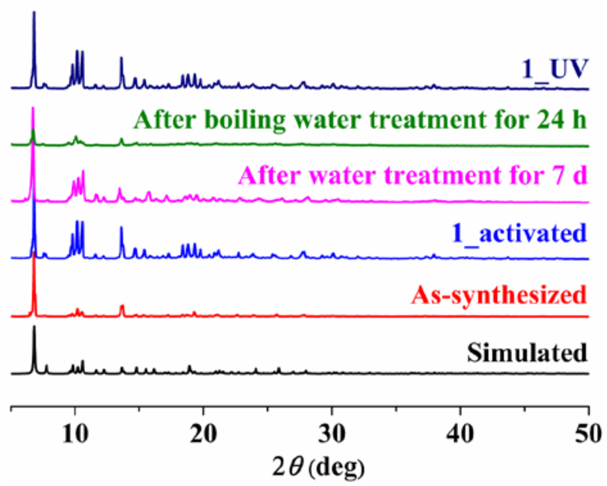

Figure S10 PXRD patterns of complex **1** upon treatment with heat, water, boiling water and UV light.

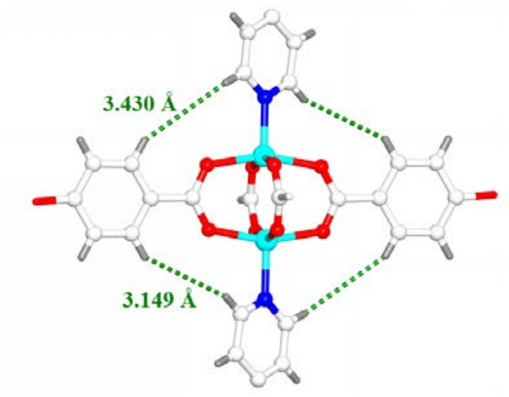

(a)

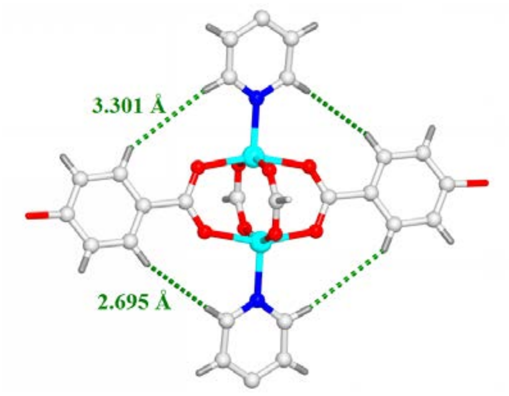

(b)

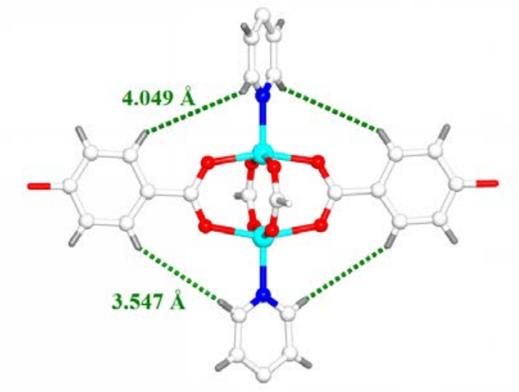

(c)

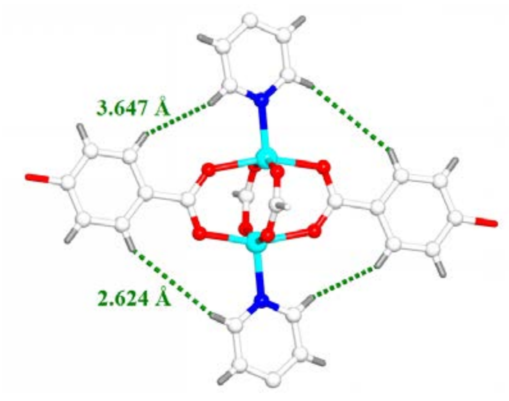

(d)

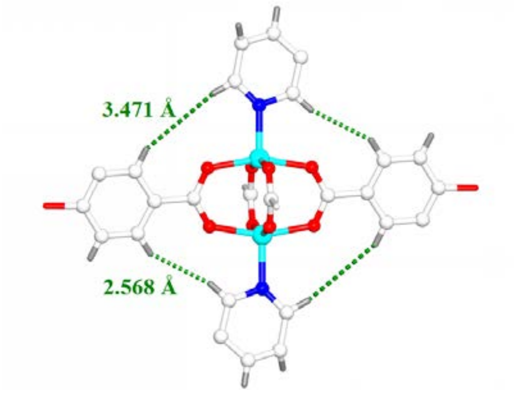

(e)

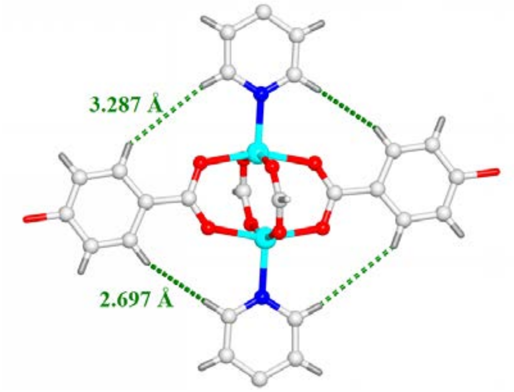

(f)

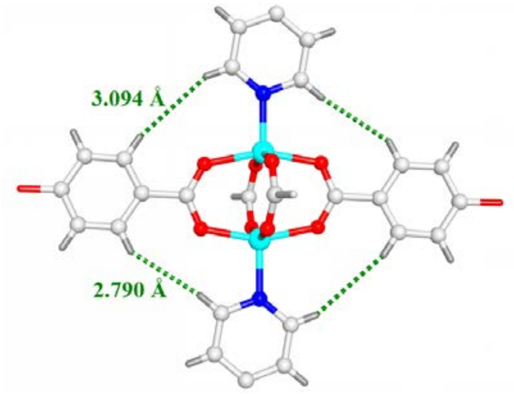

(g)

Figure S11 Distances of adjacent H atoms of the Zn_2-paddle-wheel SBUs: (a) – (f),[S1-S5] (g) this work.

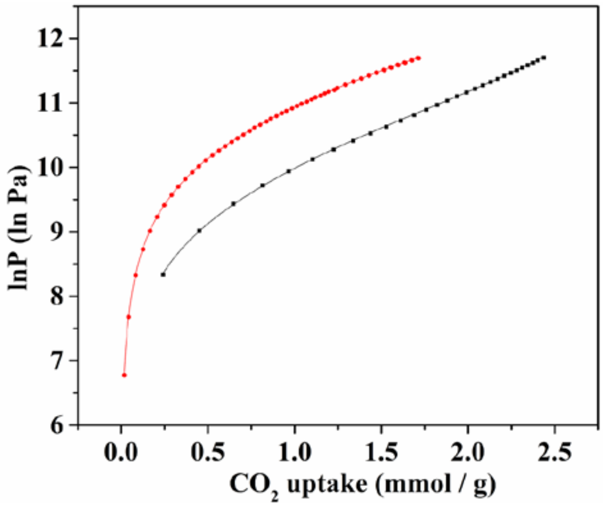

(a)

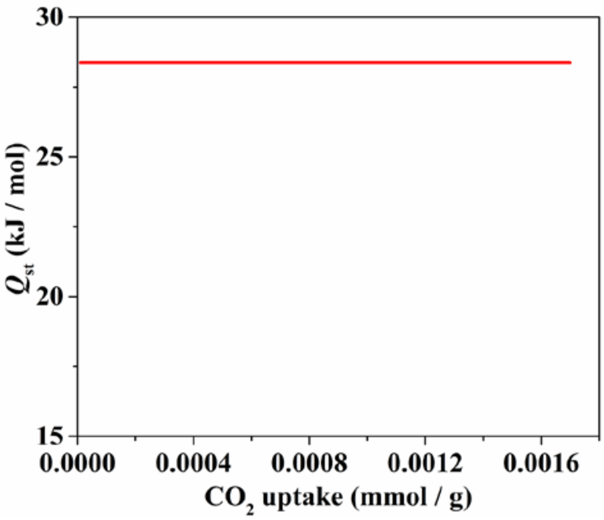

(b)

Figure S12 (a) Virial expression fitting graphs for CO_2 adsorption data for **1** at 273K (black) and 298K (red). (b) Isosteric heat of CO_2 adsorption for **1**.

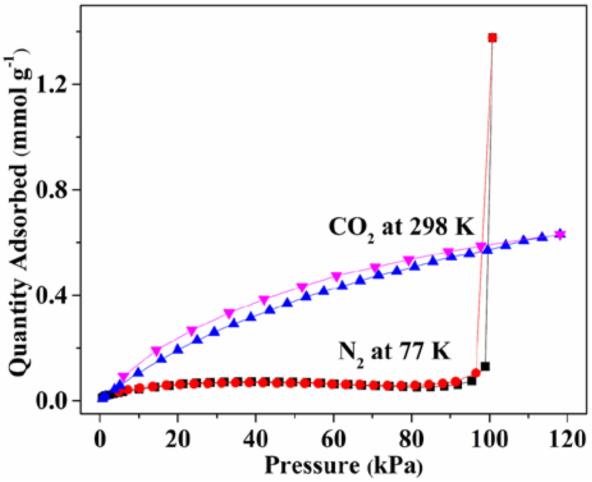

Figure S13 N_2 sorption (77 K) and CO_2 sorption (298 K) for boiling treatment samples of **1**.

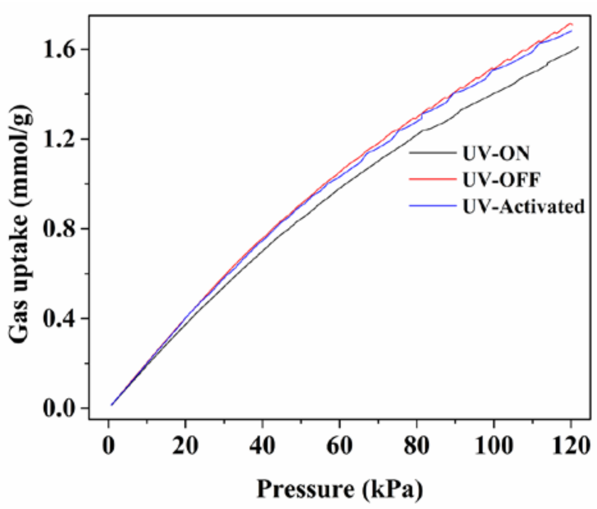

(a)

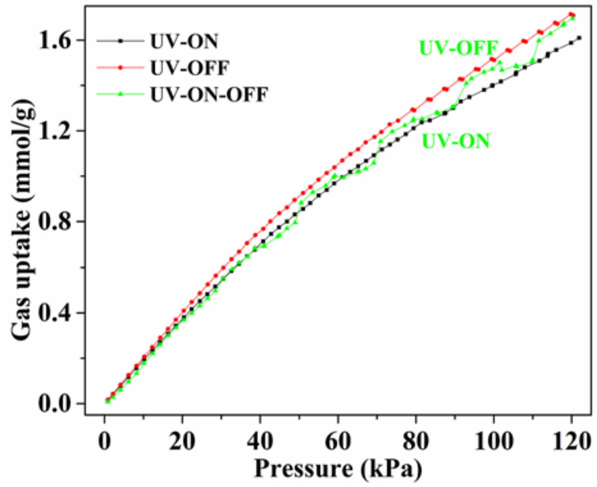

(b)

Figure S14 (a) Comparison of the CO_2 uptake at different experimental conditions. (b) Photoswitching experiment of a different batch of sample.

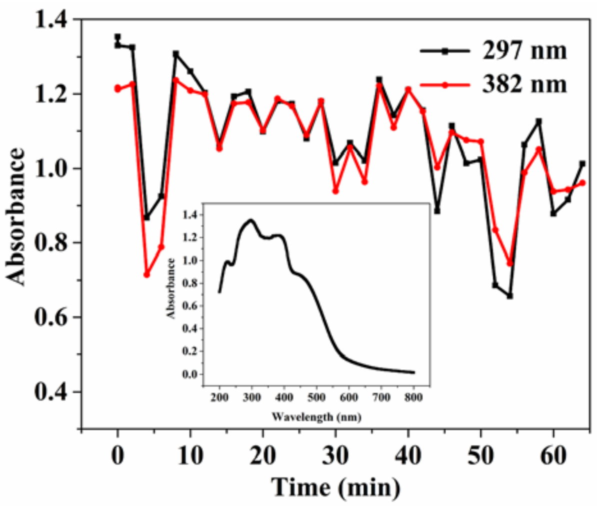

Figure S15 UV-vis spectra under UV from 0 to 60 min.

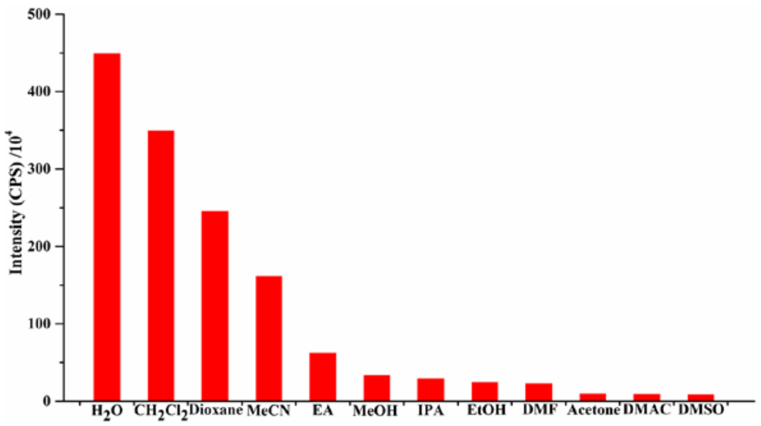

Figure S16 Emission intensity of **1** at different solvents.

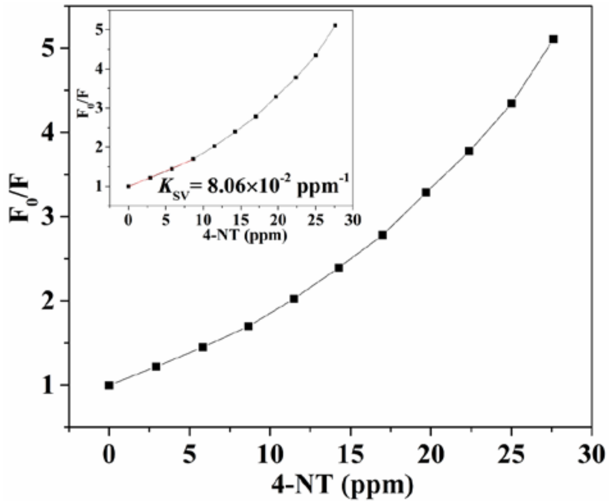

Figure S17 Stern-Volmer plot of F_0/F vs. 4-NT concentration in aqueous solution for **1**.

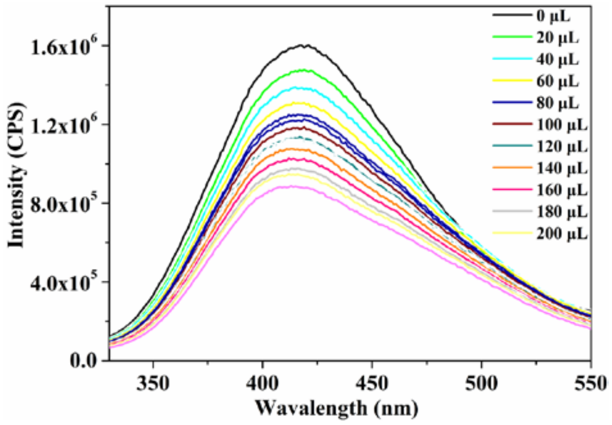

(a)

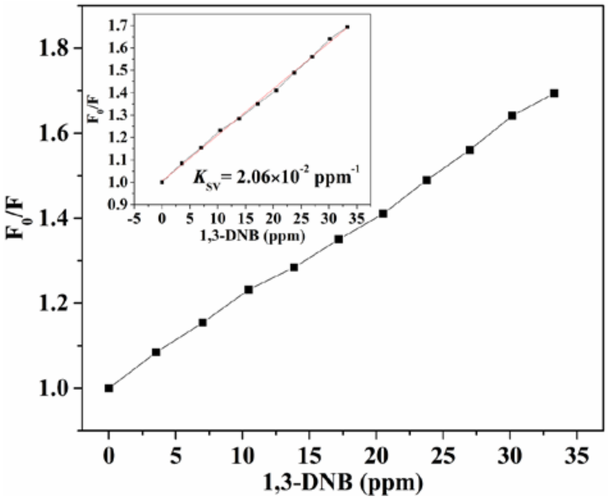

(b)

Figure S18 (a) Emission spectra of **1** at different 1,3-DNB concentrations. (b) Stern-Volmer plot of F_0/F vs. 1,3-DNB concentration in aqueous solution for **1**.

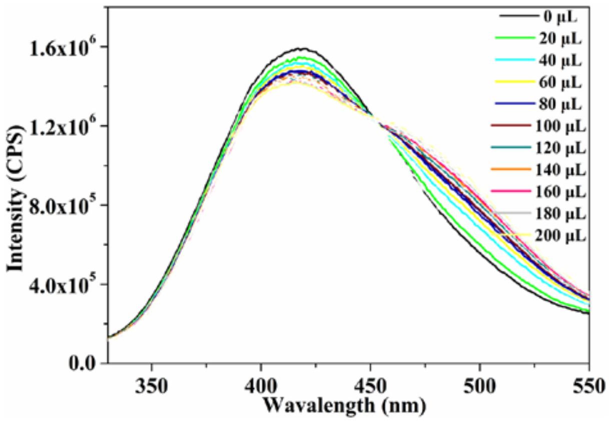

(a)

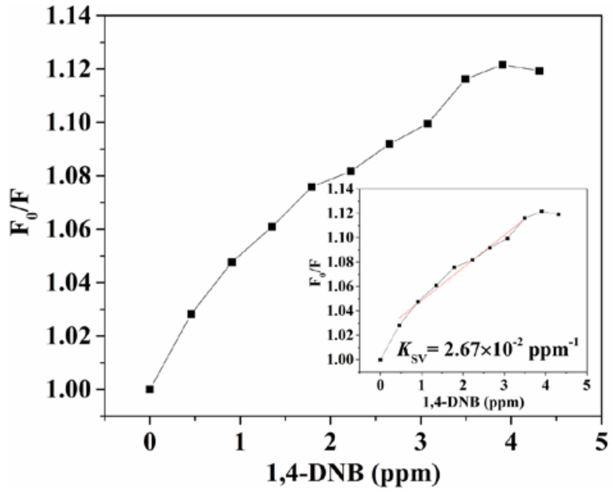

(b)

Figure S19 (a) Emission spectra of **1** at different 1,4-DNB concentrations. (b) Stern-Volmer plot of F_0/F vs. 1,4-DNB concentration in aqueous solution for **1**.

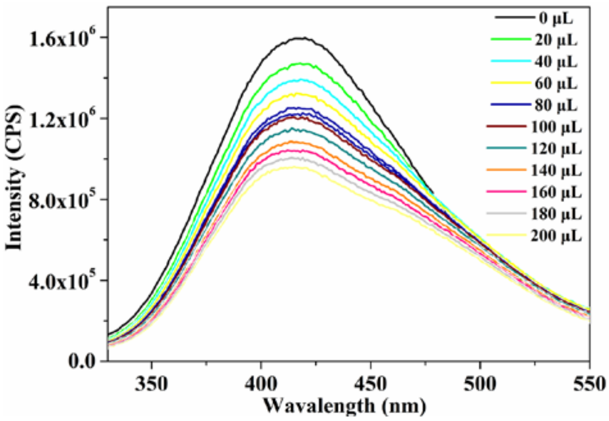

(a)

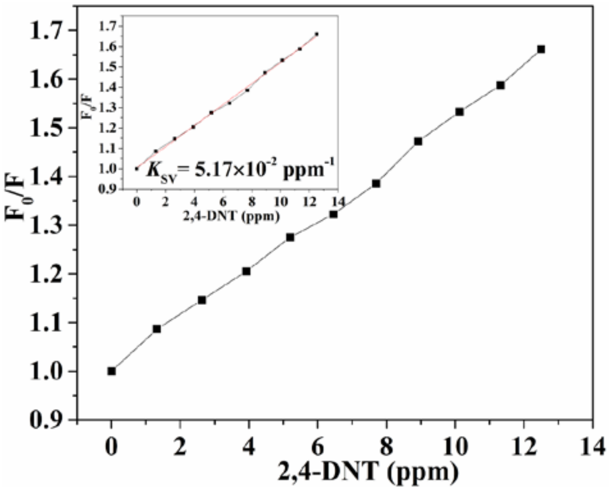

(b)

Figure S20 (a) Emission spectra of **1** at different 2,4-DNT concentrations. (b) Stern-Volmer plot of F_0/F vs. 2,4-DNT concentration in aqueous solution for **1**.

Reference:

S1 S. Pramanik, C. Zheng, X. Zhang, T, J. Emge and J. Li, *J. Am. Chem. Soc.*, 2011, **133**, 4153.

S2 M. Kondo, Y. Irie, M. Miyazawa, H. Kawaguchi, S. Yasue, K. Maeda, F. Uchida, *J. Organomet. Chem.*, 2007, **692**, 136.

S3 Y. Ge, N.-Y. Li, W. Ma, D. Liu and X.-Y. Tang, *CrystEngComm*, 2014, **16**, 10949.

S4 J. Li, Y. Peng, H. Liang, Y. Yu, B. Xin, G. Li, Z. Shi and S. Feng, *Eur. J. Inorg. Chem.*, 2011, **17**, 2712.

S5 M. Y. Masoomi, K. C. Stylianou, A. Morsali, P. Retailleau and D. Maspoch, *Cryst. Growth Des.*, 2014, **14**, 2092.
